# Clinical evaluation of augmented reality-based 3D navigation system for brachial plexus tumor surgery

**DOI:** 10.1186/s12957-023-03288-z

**Published:** 2024-01-17

**Authors:** Xuanyu Zhao, Huali Zhao, Wanling Zheng, Andreas Gohritz, Yundong Shen, Wendong Xu

**Affiliations:** 1 Department of Hand and Upper Extremity Surgery, Jing’an District Central Hospital, Branch of Huashan Hospital, Fudan University, Shanghai, China; 2 Department of Hand Surgery, Huashan Hospital, Fudan University, Shanghai, China; 3 Department of Radiology, Jing’an District Central Hospital, Branch of Huashan Hospital, Fudan University, Shanghai, China; 4 Department of Plastic, Reconstructive, Aesthetic and Hand Surgery, University Hospital Basel, University of Basel, Basel, Switzerland; 5 The National Clinical Research Center for Aging and Medicine, Fudan University, Shanghai, China; 6 Institute of Brain Science, State Key Laboratory of Medical Neurobiology and Collaborative Innovation Center for Brain Science, Fudan University, Shanghai, China; 7 Research Unit of Synergistic Reconstruction of Upper and Lower Limbs after Brain Injury, Chinese Academy of Medical Sciences, Beijing, China

**Keywords:** Augmented reality, Brachial plexus, Head-mounted display, MRI, Navigation system, Tumor

## Abstract

**Background:**

Augmented reality (AR), a form of 3D imaging technology, has been preliminarily applied in tumor surgery of the head and spine, both are rigid bodies. However, there is a lack of research evaluating the clinical value of AR in tumor surgery of the brachial plexus, a non-rigid body, where the anatomical position varies with patient posture.

**Methods:**

Prior to surgery in 8 patients diagnosed with brachial plexus tumors, conventional MRI scans were performed to obtain conventional 2D MRI images. The MRI data were then differentiated automatically and converted into AR-based 3D models. After point-to-point relocation and registration, the 3D models were projected onto the patient’s body using a head-mounted display for navigation. To evaluate the clinical value of AR-based 3D models compared to the conventional 2D MRI images, 2 senior hand surgeons completed questionnaires on the evaluation of anatomical structures (tumor, arteries, veins, nerves, bones, and muscles), ranging from 1 (strongly disagree) to 5 (strongly agree).

**Results:**

Surgeons rated AR-based 3D models as superior to conventional MRI images for all anatomical structures, including tumors. Furthermore, AR-based 3D models were preferred for preoperative planning and intraoperative navigation, demonstrating their added value. The mean positional error between the 3D models and intraoperative findings was approximately 1 cm.

**Conclusions:**

This study evaluated, for the first time, the clinical value of an AR-based 3D navigation system in preoperative planning and intraoperative navigation for brachial plexus tumor surgery. By providing more direct spatial visualization, compared with conventional 2D MRI images, this 3D navigation system significantly improved the clinical accuracy and safety of tumor surgery in non-rigid bodies.

**Supplementary Information:**

The online version contains supplementary material available at 10.1186/s12957-023-03288-z.

## Introduction

The anatomy of the brachial plexus region is complex, with major structures such as nerves and blood vessels overlapping and crossing each other [1]. Compression of normal organs and neovascularization by brachial plexus tumors adds further complexity to this region, making the management of these tumors challenging for surgeons [2, 3]. Current surgical principles advocate for preserving nearby functional nerves while attempting to completely resect the tumor, which requires personalized and high-quality surgical plans based on patient-specific anatomy before surgery, as well as precise navigation during surgery [4].

With the improvement of MRI technology, biomedical images of patients have become clearer and more accurate, which has made it closely related to clinical diagnosis and treatment of tumors [5, 6]. Although MRI images can be reconstructed into 3D images, they are currently mostly presented in 2D form, and there is still a gap between conventional flat displays and the 3D structures of the human body [7]. The lack of 3D spatial representation limits the further application value of MRI in preoperative planning and intraoperative navigation of tumor surgery.

As a novel technology that can visualize 3D biomedical data, with the improvement of related software and equipment, various augmented reality (AR)-based 3D imaging systems have been demonstrated to be safe and efficient in clinical application [8], providing a novel stereotactic navigation mode [9, 10]. After reconstructing MRI data into 3D models [11], the 3D holographic models can be projected onto the surgical region within the surgeon’s field of view using an AR-based tracking camera integrated head-mounted display (HMD) [12]. The use of these 3D visualizations is expected to improve the understanding of precise tumor locations and aid in the assessment of vital anatomical structures, including arteries, veins, and nerves [13, 14].

Currently, AR-based 3D navigation systems are mainly explored in head and spine tumor surgery [15–17]. These rigid body structures have stable morphology which facilitates convenient reconstruction and registration of AR-based 3D holographic models for defining optimal surgical strategies [18]. For non-rigid bodies such as the brachial plexus, the location of anatomical structures varies with the patient’s body position [10]. To date, no studies have investigated the clinical safety and accuracy of AR-based 3D navigation systems for brachial plexus tumors.

In this study, for the first time, we applied AR-based 3D navigation systems in the surgical treatment of brachial plexus tumors, with the surgical goal of preserving the surrounding nerves and blood vessels while completely resecting the tumor. Surgeons were asked to evaluate this AR-based 3D navigation system in brachial plexus tumors and report its potential added value which to our knowledge has never been investigated.

## Materials and methods

### Study design

Eight patients diagnosed with brachial plexus tumors and surgically treated at our center between May 2022 and May 2023 were selected as study subjects. Inclusion criteria were as follows: (1) patients with brachial plexus tumor requiring surgery, (2) preoperative needle biopsy and postoperative pathological reports confirmed schwannoma, (3) no tumor resection surgery prior to our procedure, and (4) patients confirmed to have no obvious osteoporosis, cervical spondylosis, history of drug allergy, and contraindications for MRI scan. Exclusion criteria were as follows: (1) patients with brachial plexus nerve tumors that could not be completely removed by surgery, (2) surgical procedures with severe nerve or vascular injuries requiring surgical repair, (3) patients under the age of 18, and (4) underlying diseases that affect the surgical procedure or patient’s prognosis.

**Fig. 1:**
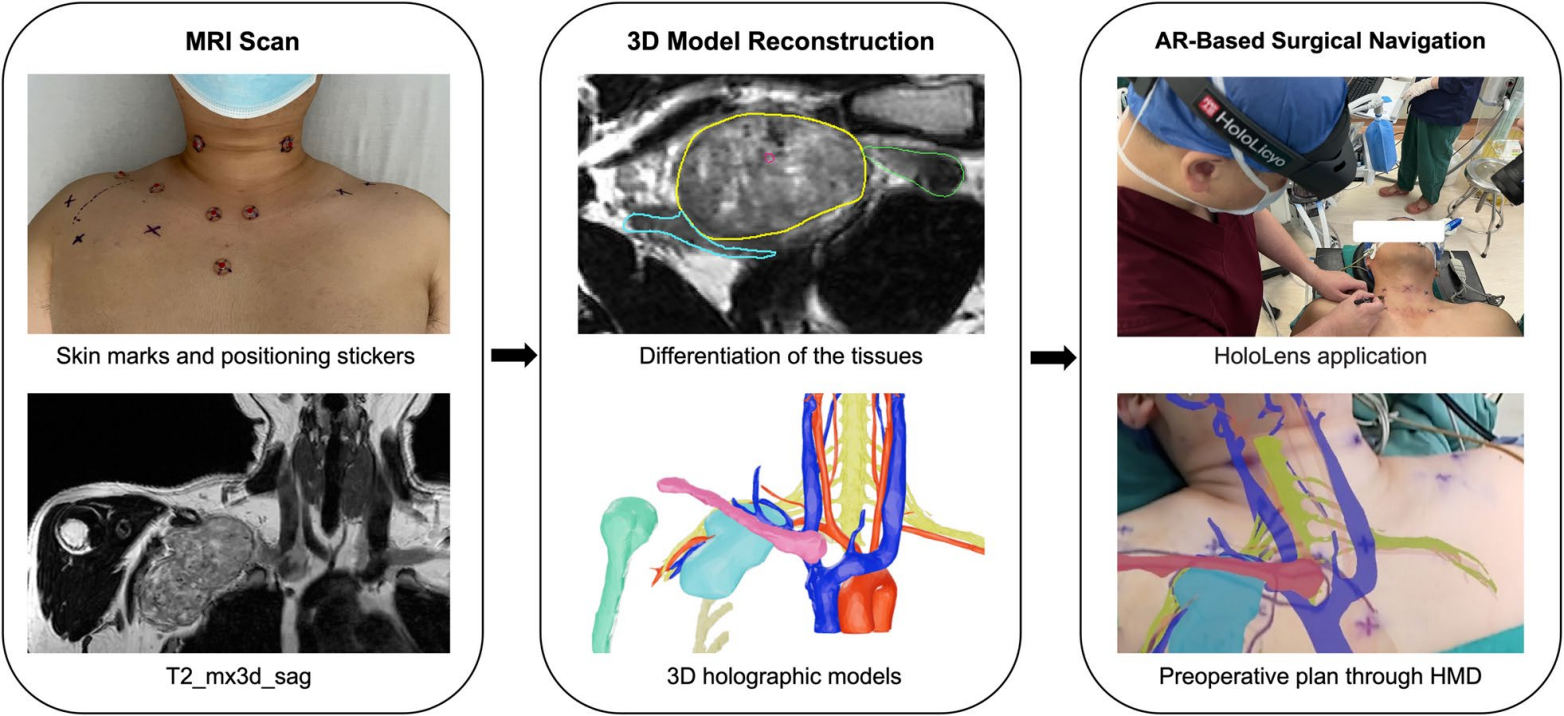
Workflow diagram illustrating the process of reconstruction and application of 3D holographic models

The main process of our AR-based 3D navigation system is as follows, as shown in Fig. 1.

- Step 1.Select identifiable anatomical landmarks on the patient’s body surface and attach MRI scan locator stickers containing contrast agent.
- Step 2.Perform both native and contrast-enhanced MRI scans of the area where the brachial plexus tumor is located.
- Step 3.Apply automatic tissue differentiation techniques to the MRI images and make necessary manual adjustments.
  Step 4.Reconstruct the processed MRI data into 3D models and upload them to the software inside the HMD.
- Step 5.Perform point-to-point relocation and registration using the chosen landmarks in the MRI scan, and project the 3D models onto the patient’s body.
  Step 6.Perform preoperative planning and intraoperative navigation following the guide of AR-based 3D holographic models.

### MRI scan

Points of anatomical landmarks were selected as location markers on the skin of the patient’s surgical region. Positioning stickers containing iodine and iron compound solution, which appear as small high-signal dots in MRI images, are attached to the skin marks as reference points for reconstruction and registration. A China United Imaging 1.5T uMR660 MRI was used in this study. During scanning, the subject was held in the supine position with a head-neck coil and a chest-abdomen coil. The scan range was from the base of the skull to the lower edge of the thoracic 4 plane.

The scan sequences were as follows: t2_mx3d_sag (FOV: 240mm, thickness/interval: 0.7/0mm, resolution: 480 × 480, TR: 1400ms, TE: 165.2ms), gre_cemra_cor_post (FOV: 350mm, thickness/interval: 1.1/0mm, Resolution: 320 × 220, TR: 4.2ms, TE: 1.8ms), and stir_mx3d_cor (FOV: 340mm, thickness/interval: 0.8/0mm, Resolution: 480 × 480, TR: 3200ms, TE: 338.5ms) (Supplementary Video 1). The first scan was t2_mx3d_sag sequence for MRI data of skin and bone. A high-pressure syringe was used to inject the contrast medium (Diamine, specification 20ml: 5.74g, total dose of 20ml, rate of 3.0ml/s) through the median elbow meridian. With the contrast medium injected, dynamic monitoring was conducted to scan the gre_cemra_cor_post sequence. One minute later, the stir_mx3d_cor sequence scan was performed, after which the image data was exported to be saved in the Dicom format.

### 3D model reconstruction

The raw MRI data was reviewed using the RadiAnt DICOM Viewer to determine the appropriate number of phases for reconstruction and to select the appropriate image sequence. Preliminary tissue differentiation was performed automatically based on the different MRI signal intensities of anatomical structures. Manual adjustments were then made through collaboration between computer scientists, radiologists, and orthopedic hand surgeons. The threshold was adjusted based on the MRI data of each patient, and the binarization preserved the voxels whose gray value was greater than or equal to the threshold in the image of the segmentation object. With the region of interest mask files, the editing functions were selected to modify the mask file to segment the target tissue boundary and remove the irrelevant tissue. The models were then polished, smoothed, and wrapped to remove the noise.

### AR-based surgical navigation

A mixed reality HMD (HoloLens; Microsoft Corp) was used for AR visualization. Data from reconstructed 3D models (*Supplementary Video* 2) were uploaded to the AR software in the HMD. After placing the surgical position of the anesthetized patient, the skin marks reconstructed in 3D models were superimposed on the anatomical landmarks of the patient selected before the MRI scan. After relocation and registration by point-based skin markers, 3D holographic models were projected onto the patient’s body, allowing surgeons to directly visualize anatomical structures through the HMD. The projection of 3D holographic models served as a guide of surgery, helping to design surgical incisions, predict the location of anatomical structures, and protect critical structures within the surgical field.

During the operation, after anatomical structures are exposed, there may be cases where the 3D models do not perfectly match the actual structures because the registration is based solely on skin markers. To ensure accurate spatial alignment of the AR projection, intraoperative real-time re-registration was performed by the surgeons to compensate for positional error between the AR models and the real anatomy.

### Questionnaire

Two hand surgeons with more than 20 years of experience in brachial plexus tumor surgery were asked to individually evaluate conventional MRI images and AR-based 3D models for each patient after surgery. They completed questionnaires assessing the quality of both imaging modalities in visualizing anatomical structures.

A rating scale from 1 to 5 was used to score the visibility of 6 anatomical structures: tumor, arteries, veins, nerves, bones, and muscles (1, strongly disagree; 2, disagree; 3, neutral; 4, agree; and 5, strongly agree). Surgeons were also asked to rate their willingness to use conventional MRI images or AR-based 3D models for preoperative planning and intraoperative decision making. In addition, during surgery, surgeons were asked to measure and report the positional error (in centimeters) between the projection position of the 3D models and intraoperative findings. This positional error is due to the fact that only preoperative skin registration was performed. After measuring the positional error, surgeons would perform intraoperative re-registration to correct this deviation. The questionnaires can be found in the Supplementary Material.

### Statistical analysis

GraphPad Prism (version 9) was used for statistical analysis. The nonparametric Wilcoxon matched-pair signed rank test was used to compare the results between conventional MRI images and AR-based 3D models. A two-tailed *p* value of less than 0.005 was considered statistically significant.

## Results

### Patient characteristics

The characteristics of the 8 patients included in this study are summarized in Table 1. The average time for the 3D model reconstruction process was 1.8 ± 0.7 h. After anesthesia and before the start of surgery, the point-to-point registration took 16 ± 5 min (mean ± standard deviation); during the surgery, the real-time re-registration took 7 ± 3 min. Besides incisional pain, no patients experienced complications related to tumor excision surgery or the use of AR-based 3D navigation, including limb motor dysfunction or sensory disturbances due to nerve damage, circulatory disturbances, or abnormal limb temperatures due to vascular damage.

**Table 1:** Clinical characteristics of 8 patients with brachial plexus tumor

Clinical characteristics	Cases (percentage)
Gender	
Male	5 (62.5%)
Female	3 (37.5%)
Tumor type	
Schwannoma	8 (100%)
Tumor location	
Roots	1 (12.5%)
Trunks	3 (37.5%)
Cords and branches	4 (50.0%)
Largest dimension	
< 5cm	6 (75.0%)
≥ 5cm	2 (25.0%)

### Evaluation of anatomical structures

**Fig. 2:**
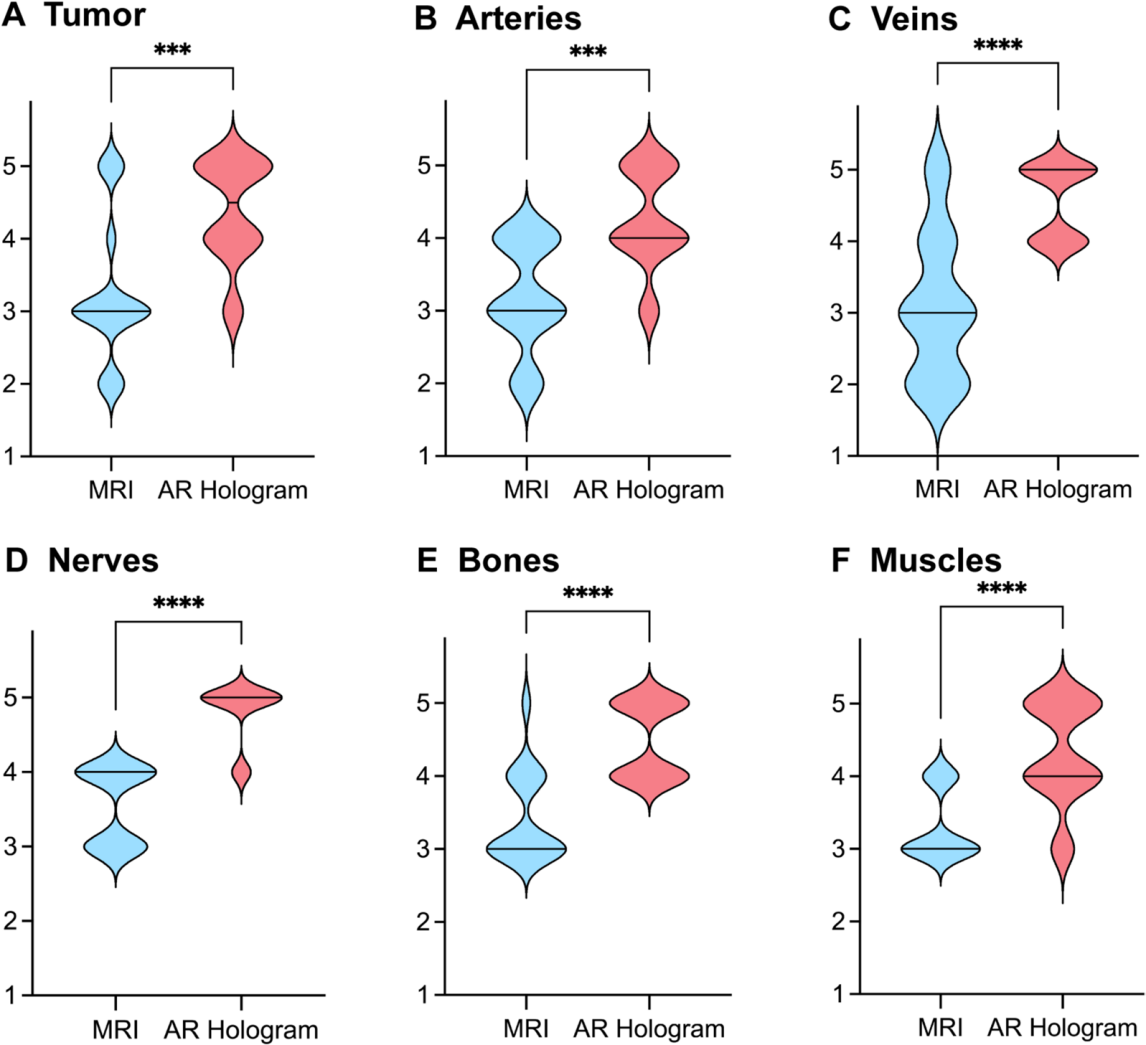
Comparison of questionnaire scores for various anatomical structures between conventional MRI images (Blue) and AR-based 3D models (Red). Two surgeons independently rated the visibility of 6 anatomical structures using a scale from 1 to 5 (1 indicating strongly disagree; 2, disagree; 3, neutral; 4, agree; and 5, strongly agree) for both conventional MRI images and AR-based 3D models. The results represent the comparison of mean scores from eight patients, with the middle lines indicating the medians

Compared with conventional MRI images, AR-based 3D holographic models were judged superior by surgeons for all anatomical structures (Fig. 2 and Table 2): tumor (MRI, 3.25 ± 1.00 vs AR hologram, 4.38 ± 0.72; *p* < 0.001), arteries (MRI, 3.19 ± 0.75 vs AR hologram, 4.25 ± 0.68; *p* < 0.001), veins (MRI, 3.13 ± 1.02 vs AR hologram, 4.69 ± 0.48; *p* < 0.001), nerves (MRI, 3.56 ± 0.51 vs AR hologram, 4.63 ± 0.50; *p* < 0.001), bone (MRI, 3.44 ± 0.63 vs AR hologram, 4.50 ± 0.70; *p* < 0.001), and muscle (MRI, 3.31 ± 0.48 vs AR hologram, 4.31 ± 0.70; *p* < 0.001).

**Table 2:** Anatomical structure assessment scores from questionnaires

Anatomical structure	Score, mean (SD)		*p* value
	Conventional MRI images	AR-based 3D models	
Tumor	3.25 (1.00)	4.38 (0.72)	< 0.001
Arteries	3.19 (0.75)	4.25 (0.68)	< 0.001
Veins	3.13 (1.02)	4.69 (0.48)	< 0.001
Nerves	3.56 (0.51)	4.63 (0.50)	< 0.001
Bones	3.44 (0.63)	4.50 (0.70)	< 0.001
Muscles	3.31 (0.48)	4.31 (0.70)	< 0.001

### Added value of 3D imaging

**Fig. 3:**
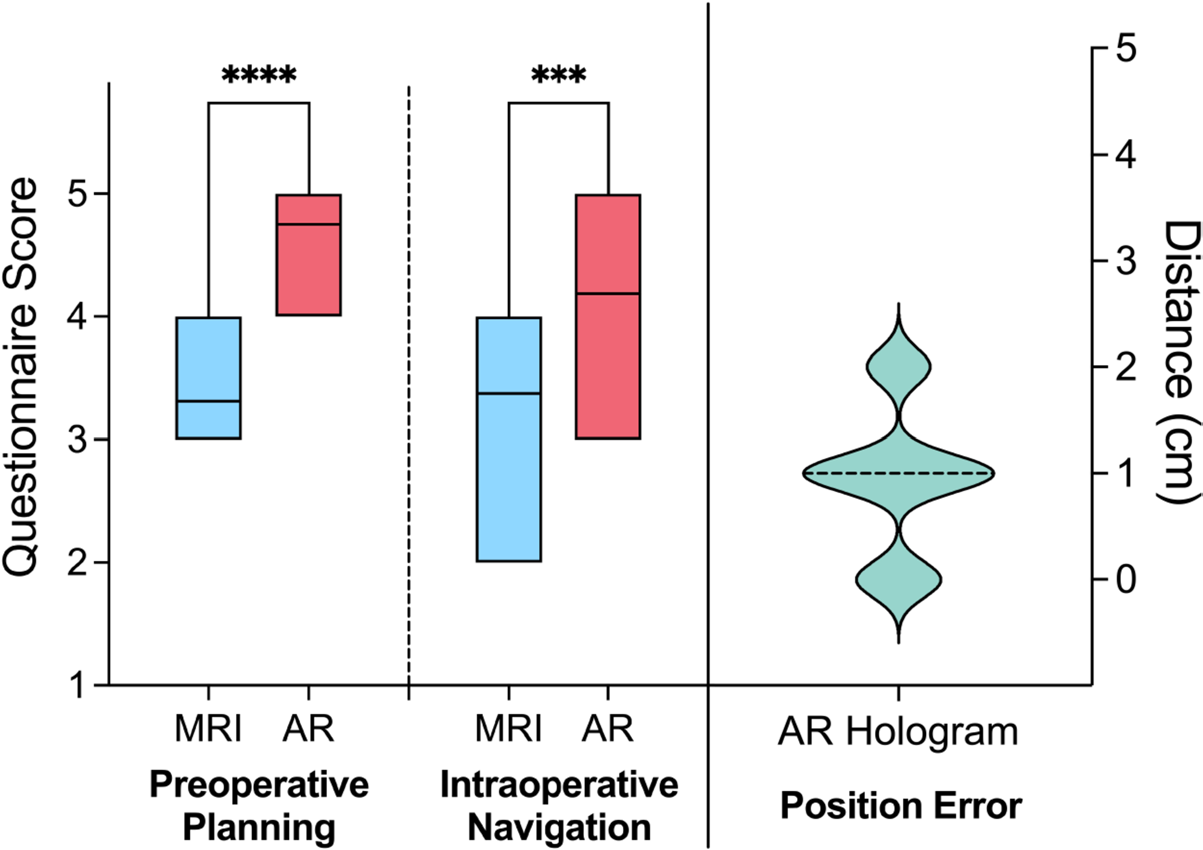
Comparison of questionnaire scores for the added value in preoperative planning and intraoperative navigation between conventional MRI images (blue) and AR-based 3D models (red). Surgeons were asked to measure the position error (cm) between 3D models and intraoperative findings (green) before re-registration during surgery. Middle lines indicate median values

AR-based 3D models were also more likely to be used in preoperative planning (MRI, 3.38 ± 0.62 vs AR hologram, 4.75 ± 0.45; *p* < 0.001) and intraoperative navigation (MRI, 3.31 ± 0.48 vs AR hologram, 4.19 ± 0.75; *p* < 0.001) for the added value (Fig. 3). The satisfaction of the surgeons with AR-based 3D models has reached 4.4 ± 0.7. The deviation distance between the location 3D models provided before re-registration and the intraoperative findings was 0.94 ± 0.68 cm on average (Fig. 3).

## Discussion

Achieving complete tumor resection while preserving the surrounding nerves and blood vessels is a major challenge in brachial plexus tumor surgery [4]. These critical neurovascular structures are often severely deformed and displaced by tumor compression. Any damage to them can lead to serious complications in limb movement or sensory function. With its high-quality soft tissue imaging capabilities, MRI has been widely used in the surgical treatment of brachial plexus tumors [1]. However, conventional 2D imaging methods are limited to providing positional information in a single plane, restricting the use of MRI data and failing to provide surgeons with a comprehensive representation of abnormal anatomical structures caused by tumors [10]. Conversion of conventional MRI data into 3D visualization can provide a comprehensive understanding of anatomical structures for the improvement of tumor resection without compromising normal functions. Several 3D imaging navigation approaches have been developed and applied for head and spine tumor surgery [11, 19, 20]. The relatively stable positioning of structures in the head and spine has allowed for the successful application of AR-based navigation systems. However, due to the instability of the positional relationships of structures in the brachial plexus region, our understanding of the effectiveness of 3D imaging in tumors in this region remains limited [21].

In this study, we introduced an innovative AR-based navigation system specifically designed for brachial plexus tumors, marking the first application of such a 3D navigation system in this region. 3D models of the patient’s surgical region were constructed based on preoperative MRI images and uploaded to AR software in the HMD. The surgical team can project 3D holographic models of the tumor, blood vessels, nerves, bones, and muscles onto the patient’s body, allowing flexible observation of different anatomical layers and angles through the HMD. It has been demonstrated that the new 3D imaging technology can fully utilize MRI image data to provide surgeons with spatially enhanced visualization of anatomical structures prior to surgery [22].

Preoperatively, the 3D models reconstructed from MRI images accurately depicted the tumor morphology, allowing for the evaluation of anatomical variations in patients and guiding the design of the most appropriate surgical incisions. These 3D models also facilitated preoperative rehearsals and contingency plans for potential complications to improve surgical preparation. Intraoperatively, the AR-based 3D models were projected onto the patient’s body through HMD, allowing simultaneous viewing of the surgical field and navigation models within the same field of view. This real-time visualization provided valuable guidance by predicting the anatomical relationship between the tumor and surrounding structures, effectively preventing potential injury to critical nerves and blood vessels. By providing real-time warnings of critical structures within the surgical field that may not be immediately visible, AR-based 3D models also simplified the challenging learning curve of brachial plexus tumor surgery, significantly improving the efficiency and safety.

To maximize the safety and effectiveness of the AR system, we performed a two-step registration process. Point-to-point registration was conducted prior to surgery, followed by real-time re-registration after the anatomical structures were exposed during surgery. These two registration processes help ensure that the position provided by the AR-based 3D model is optimally aligned with the location of the tumor, thereby effectively preventing complications associated with AR technology. Given the benefits of AR technology in increasing precision and safety during surgery, the time spent in registration could be compensated by a more streamlined and efficient surgical procedure. The additional ten minutes spent on re-registration during surgery is acceptable when weighed against the risk of neurovascular damage.

The HoloLens HMD used in this study costs $3500 in dollars, while the cost of commercially available 3D printing is typically $500 in dollars [10]. The AR HMD is a one-time investment whose value is maximized through repeated use in patients. Its application in eight patients included in this study has already demonstrated its cost-effectiveness. Compared to 3D printing [10, 23], which requires a fabrication time of 4 to 5 days, AR reconstruction takes only about 2 h and requires no material costs. In addition, AR-based 3D models can be seamlessly integrated with the actual patient anatomy, creating a mixed-reality environment that can be directly used as a navigation system during surgery—an advantage that cannot be attained with 3D printing. The potential for economic and time-saving efficiency positions AR-based 3D imaging as a highly promising technology for clinical applications.

This study has several limitations. Only a limited number of surgeons participated in the evaluation of early experiences with our AR-based 3D navigation system. Only 8 patients with schwannoma were included because it is the most common benign tumor of the brachial plexus, and it can be completely resected by surgery. Further confirmatory clinical studies involving a larger number of patients, surgeons, and institutions are essential for widespread adoption, providing a broader and more representative data set for more comprehensive analysis and validation. High-quality MRI images are critical to obtaining well-rendered 3D reconstruction models. To ensure accurate reconstruction of the skin landmarks, each patient underwent more than 1 h of MRI scans. Therefore, further research is needed to standardize the optimal MRI sequences, slice thickness, and timing of contrast enhancement. Despite the use of point-based skin markers for registration and manual realignment for re-registration, achieving a stable 3D hologram still required precise patient positioning and minimal head movement by the surgeon to reduce mismatch and drift. This aspect is expected to improve with the advancement of tracking algorithms in the future.

## Conclusions

This study provided the first effective evidence that the 3D navigation system improves the clinical accuracy and safety of brachial plexus tumor surgery. AR-based 3D holographic models showed better visualization of anatomical structures than conventional MRI images, thereby improving preoperative planning and intraoperative navigation, making it a valuable adjunct to surgical treatment that deserves wider clinical application.

### Supplementary Information

**Additional file 1.**

**Additional file 2.**

**Additional file 3.**

## Data Availability

The datasets used or analyzed during the current study are available from the corresponding authors on reasonable request.
