# Supplementary material for: Clinical evaluation of augmented reality-based 3D navigation system for brachial plexus tumor surgery
Source: World J Surg Oncol. 2024 Jan 17;22:20. doi: 10.1186/s12957-023-03288-z (PMC10792838; doi:10.1186/s12957-023-03288-z)
Supplement: Supplementary file 3 — Additional file 3. [file 12957_2023_3288_MOESM3_ESM.pdf]

Questionnaire of conventional MRI images in surgery for brachial plexus tumor

The tumor can be well assessed based on MRI images.	1 - 2 - 3 - 4 - 5
The arterial structures can be well assessed based on MRI images.	1 - 2 - 3 - 4 - 5
The venous structures can be well assessed based on MRI images.	1 - 2 - 3 - 4 - 5
The nervous structures can be well assessed based on MRI images	1 - 2 - 3 - 4 - 5
The bones structures can be well assessed based on MRI images.	1 - 2 - 3 - 4 - 5
The muscular structures can be well assessed based on MRI images.	1 - 2 - 3 - 4 - 5
MRI images prepares me for surgery.	1 - 2 - 3 - 4 - 5
I expect no complicating events based on MRI images.	1 - 2 - 3 - 4 - 5
I am likely to consult MRI images in the preoperative planning.	1 - 2 - 3 - 4 - 5
I am likely to consult MRI images during surgery.	1 - 2 - 3 - 4 - 5

1, strongly disagree; 2, disagree; 3, neutral; 4, agree; and 5, strongly agree

Comments on conventional MRI images:

____

____

____

Questionnaire of AR-based 3D holographic models in surgery for brachial plexus tumor

The tumor can be well assessed based on 3D models.	1 - 2 - 3 - 4 - 5
The arterial structures can be well assessed based on 3D models.	1 - 2 - 3 - 4 - 5
The venous structures can be well assessed based on 3D models.	1 - 2 - 3 - 4 - 5
The nervous structures can be well assessed based on 3D models.	1 - 2 - 3 - 4 - 5
The bones structures can be well assessed based on 3D models.	1 - 2 - 3 - 4 - 5
The muscular structures can be well assessed based on 3D models.	1 - 2 - 3 - 4 - 5
3D models prepare me for surgery.	1 - 2 - 3 - 4 - 5
I expect no complicating events based on 3D models.	1 - 2 - 3 - 4 - 5
I am likely to consult 3D models in the preoperative planning.	1 - 2 - 3 - 4 - 5
I am likely to consult 3D models during surgery.	1 - 2 - 3 - 4 - 5
3D models are of additional value to the current imaging techniques in the preoperative planning.	1 - 2 - 3 - 4 - 5
3D models are of additional value to the current imaging techniques in the intraoperative navigation.	1 - 2 - 3 - 4 - 5
The position error (cm) between 3D models and intraoperative finding.	0 - 1 - 2 - 3 - 4 - 5

1, strongly disagree; 2, disagree; 3, neutral; 4, agree; and 5, strongly agree

Comments on AR-based 3D holographic models:

____

____

____
